# *Specific* Bioelectrical Vector Reference Values for Italian Adults: A Multicentre Study

**DOI:** 10.3390/jfmk11010081

**Published:** 2026-02-17

**Authors:** Federica Frau, Eduardo Pizzo Junior, Valeria Succa, Silvia Stagi, Federica Moro, Francesco Sguaizer, Cristian Petri, Antonio Paoli, Gabriele Mascherini, Pascal Izzicupo, Simona Bertoli, Luisa Gilardini, Luca Cavaggioni, Emanuele Cereda, Francesco Campa, Margherita Micheletti Cremasco, Stefania Toselli, Elisabetta Marini

**Affiliations:** 1 Department of Life and Environmental Sciences, University of Cagliari, 09042 Monserrato, Italy; eduardo.pizzojunior@unica.it (E.P.J.); valerias@unica.it (V.S.); silviastagi89@gmail.com (S.S.); 2 Department for Life Quality Studies, University of Bologna, 47921 Rimini, Italy; federica.moro10@unibo.it (F.M.); stefania.toselli@unibo.it (S.T.); 3 Department of Life Science and Systems Biology, University of Torino, 10123 Torino, Italy; francesco.sguaizer@unito.it (F.S.); margherita.micheletti@unito.it (M.M.C.); 4 A.C.F. Fiorentina S.r.l., 50137 Florence, Italy; cristian.petri25@gmail.com; 5 Department of Biomedical Sciences, University of Padua, 35131 Padua, Italy; antonio.paoli@unipd.it (A.P.); francesco.campa@unipd.it (F.C.); 6 Department of Experimental and Clinical Medicine, University of Florence, 50134 Florence, Italy; gabriele.mascherini@unifi.it; 7 Department of Medicine and Aging Sciences, University of Chieti-Pescara, 66100 Chieti, Italy; pascal.izzicupo@unich.it; 8 Department of Food, Environmental and Nutritional Sciences, University of Milan, 20133 Milan, Italy; simona.bertoli@unimi.it; 9 Laboratory of Nutrition and Obesity Research, IRCCS Istituto Auxologico Italiano, 20145 Milan, Italy; l.gilardini@auxologico.it (L.G.); cavaggioni.luca@gmail.com (L.C.); 10 Department of Theoretical and Applied Sciences, eCampus University, 22060 Novedrate, Italy; 11 Nutrition and Dietetics Service, Fondazione IRCCS Policlinico San Matteo, 27100 Pavia, Italy; e.cereda@smatteo.pv.it

**Keywords:** body composition, bioelectrical impedance vector analysis, BIVA, athletes, malnutrition

## Abstract

**Objective:** Since *specific* bioelectrical reference values for Italian adults are lacking, this study aims to define *specific* values and test their suitability in pathological cases and athletes. **Methods:** A sample of 1049 Italian individuals (441 men, 608 women) aged 30–65 years was considered. Competitive athletes (bodybuilding, streetlifting and tennis) were identified within the general sample, and an independent group of individuals with obesity or anorexia nervosa was analyzed for comparison. Anthropometric (weight, kg; stature, mid-upper arm, waist and calf circumferences; cm) and bioelectrical (resistance and reactance, at 50 kHz) variables were taken. Resistivity, (R*sp*, Ωcm), reactivity (Xc*sp*, Ωcm), impedivity (Z*sp*, Ωcm) and phase angle (PhA, °) were calculated. Two-way ANOVA and Hotelling’s T^2^ test were applied to assess group differences. These data were then pooled with existing datasets to create a comprehensive reference for individuals aged 18 to 100 years. **Results:** The *specific* bioelectrical variables were: R*sp* = 352.3 ± 55.5, Xc*sp* = 41.8 ± 9.1, PhA = 6.8 ± 1.0, r (R*sp*, Xc*sp*) = 0.67 (men); R*sp* = 384.9 ± 71.2, Xc*sp* = 40.7 ± 9.4, PhA = 6.1 ± 1.0, r (R*sp*, Xc*sp*) = 0.72 (women). Men showed higher PhA values (*p* < 0.001), reflecting higher muscle mass and quality, and shorter vectors (*p* < 0.001), indicative of lower relative fat mass (FM%), than women. Advancing age was associated with lower PhA and longer vectors (*p* < 0.001). Bioelectrical vectors of individuals with obesity or anorexia nervosa were outside the 95% variability, indicating abnormal values of FM%, whereas those of athletes fell within the lower left quadrant. **Conclusions:** The *specific* tolerance ellipses for the Italian adult population fill a gap in the existing literature, providing essential new tools for evaluating body composition in clinical and sports settings, and for comparative analyses.

## 1. Introduction

Body composition, that is, the absolute and relative amount and the distribution of body compartments, plays a significant role in health and disease and represents a key determinant of nutritional status [1]. It changes in relation to age, sex, and lifestyle, particularly diet and physical activity. Alterations in body composition are linked to widespread and increasing diseases, such as obesity and sarcopenia, that present relevant public health challenges worldwide. According to World Health Organization data, in 2022, 43% of adults were overweight and 16% were obese [2]. At the same time, population ageing has led to a growing burden of age-related conditions, such as sarcopenia or sarcopenic obesity, characterized by changes in body composition and strength [3,4]. Beyond clinical settings, body composition is of particular relevance in sports science, as it is influenced by sports-specific training and is related to injury risk, athletic performance, and outcomes [5,6]. Body composition of athletes is characterized by high amounts of muscle mass and low relative fat mass. These features underscore the need for tailored body composition assessment and interpretation within athletic populations and/or within the broader physiological variability of the general population. Overall, the screening and monitoring of body composition is crucial across multiple research fields and has potential applications in routine clinical practice, sports science, nutrition studies, and public health [1,7]. These applications generally involve large numbers of individuals and/or are conducted in non-laboratory, real-world settings. In such contexts, the most accurate methods, such as dual-energy X-ray absorptiometry (DXA), magnetic resonance imaging and computed tomography, are difficult or even impossible to apply. Bioimpedance analysis (BIA) represents a suitable alternative, as it is portable, relatively easy-to-use, and cost-effective [8].

Conventional BIA is based on the application of regression equations [9]. However, such procedure relies on assumptions regarding body hydration and body proportions that may not be respected, potentially leading to estimation errors. For this reason, the direct analysis of raw data, such as phase angle [10,11] or bioelectrical impedance vector analysis (BIVA) [12,13] has gained increasing attention in recent decades [14,15].

Phase angle is considered an indicator of cell mass and membrane integrity, particularly of skeletal muscle mass, and of body fluids distribution.

Vector analysis offers a wider perspective, as it is based on the contextual analysis of phase angle and vector length. Standardized resistance (R) and reactance (Xc) are the coordinates of the vector on the RXc graph, with the length corresponding to impedance (Z, Ω), and the slope to phase angle (PhA, °). In the classic BIVA procedure, as originally proposed by Piccoli et al. [12], both R and Xc are standardized for stature. In the *specific* BIVA (*sp*BIVA) approach, following the principles of Ohm’s second law, R and Xc are standardized for both stature and body cross-sectional areas [13]. In both approaches, the analysis is based on tolerance and confidence ellipses. Tolerance ellipses represent the percentile distribution of bioelectrical values, shown as three concentric ellipses, depicting 50, 75 and 95% of the values of the reference population. Body composition is interpreted based on the position of individual or mean vectors within sex-specific ellipses. The interpretation of the major axis, which is mainly determined by impedance values, differs between the classic and *specific* approaches. In classic BIVA, a longer vector is related to lower total body water, whereas in *specific* BIVA it corresponds to a higher percentage of fat mass (FM%), as validated through comparison with DXA [13]. The vector’s position along the minor axis depends on PhA, which is unaffected by standardization and hence similarly interpreted in both classic and *specific* BIVA. Vectors shifting toward the left, i.e., with high PhA values, are associated with low extracellular/intracellular water ratio (ECW/ICW), and with high body cell mass and skeletal muscle mass, whereas vectors toward the right indicate the opposite pattern [13,16].

Given the variability of body composition across sex, population, and the life stage, several tolerance ellipses have been proposed to represent populations with distinct characteristics. Most of the reference values currently available in the literature are limited to PhA (e.g., [17]). As to BIVA, a recent scoping review by Serafini et al. [18] highlighted the availability of diverse reference values, encompassing a wide range of populations, including the general population, clinical samples, and athletes. However, most of them pertain to classic BIVA, whereas *specific* reference values for the general population are available for Italian-Spanish young adults (18–30 years) [19], Italian older people (65–100 years) [16], and US young adults (21–49 years) [13]. Middle-aged adults are not represented by *specific* tolerance ellipses. Although the need for accurate age- or population-matched comparisons is not an absolute requirement but depends on the aim of the study, the absence of such data limits the representativeness of existing *specific* reference values in the adult general population.

This multicenter study aims to address the current lack of references by defining *specific* bioelectrical reference values for Italian adults, and to test their suitability for evaluating the distinctive body composition features observed in pathological cases and athletes.

## 2. Materials and Methods

### 2.1. The Sample

**Figure 1:**
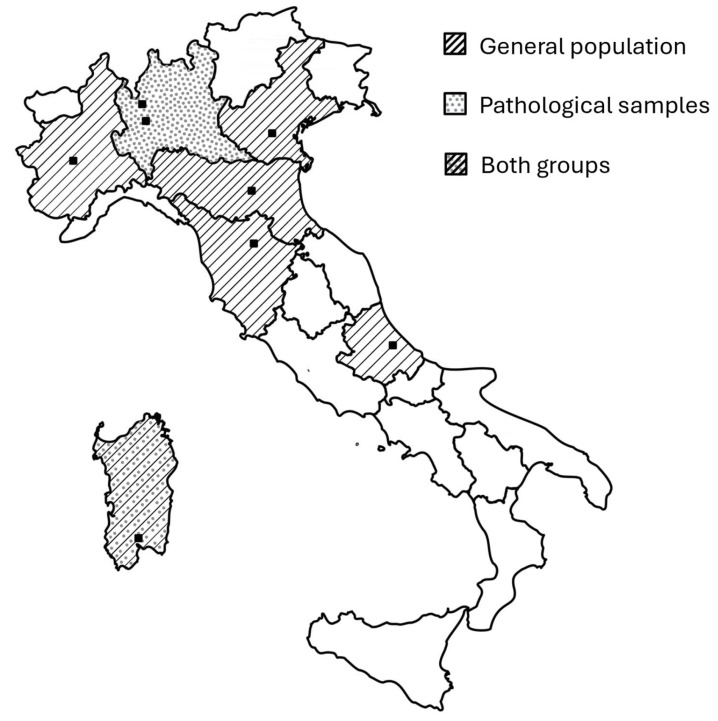
Geographical distribution of centers and participants involved across Italian regions. Black squares indicate the precise location of the centers involved.

**Figure 2:**
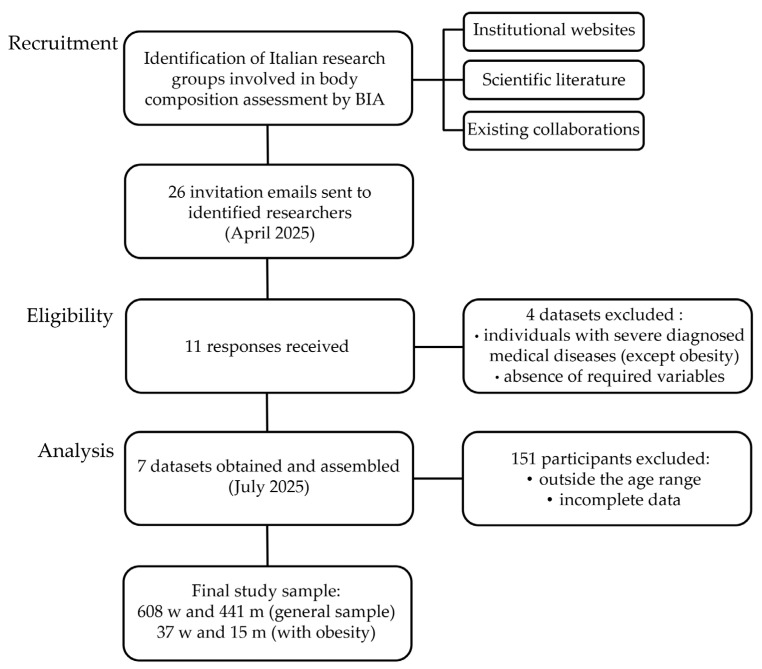
Flowchart of the data collection phases. Legend: BIA: bioelectrical impedance analysis; M: men; W: women.

The study was designed as a multicenter, retrospective and cross-sectional investigation involving eight Italian universities and two research centers (Figure 1). Data collected across the participating centers within the past five years were pooled for this study. Dataset assembly involved recruitment of research groups, eligibility assessment, data collection and analysis, and subsequent case exclusions (Figure 2).

Participants were recruited via convenience sampling from university staff, student communities, public health promotion events, and local sports clubs across the involved centers. The inclusion criteria were applied to capture a heterogeneous sample from the general population and referred to the following: age 30–65 years; provision of written informed consent; absence of pregnancy and lactation, chronic diseases, limb amputations, or implanted electrical devices. A total of 1049 participants, of which 441 men and 608 women—aged 48.8 ± 10.9 and 50.4 ± 10.2 years, respectively—were included in the analysis: 416 were enrolled at the center of Cagliari, 201 in Bologna, 187 in Turin, 162 in Florence, 68 in Padua, and 15 in Chieti.

A sub-sample of 16 competitive athletes, as defined following the classification of McKinney et al. [20], was selected from the whole sample. These athletes practiced bodybuilding [21], tennis [22] or streetlifting [23] for at least two years prior to measurement and were engaged in ≥6 h of training per week. In particular, tennis and streetlifting athletes regularly participated in competitive events at regional and national events, whereas bodybuilding athletes competed occasionally.

Furthermore, an independent sample of individuals with pathologies, with similar age, was analyzed in comparison with the variability of the general population: 52 patients with obesity, recruited at the Laboratory of Nutrition and Obesity Research, IRCCS Istituto Auxologico Italiano (Milan, Italy), and 4 patients with anorexia nervosa, including 2 young women, retrieved from the literature [24]. Both samples were analyzed using the same bioelectrical impedance device (manufactured by Akern) and pre-assessment conditions (see Section 2.2) and following the same approach for *specific* BIVA-derived variables (see Section 2.3).

All procedures were conducted in accordance with the Declaration of Helsinki for studies involving human subjects [25]; all the participants were informed about the study procedures and gave the written consent to participate. The study was approved by the Ethics Committee of centers involved in the study: Ethics Committee of the University of Cagliari (approval 0312892, 13 November 2025); Bioethics Committee of the University of Bologna (approvals 0121439, 20 May 2022, and 0157696, 6 June 2024; Bioethics Committee of the University of Torino (approval n°0640369, 7 December 2023); Ethics Committee of the University of Firenze (approval 0259279); Ethics Committee for Clinical Drug Trials—Local Health Authority of Pescara (approval n°1070 of 24 October 2013); Ethics Committee for Research Involving Human Subjects—Department of Biomedical Sciences of University of Padova (approval n° HEC-DSB/02-2023 of 31 July 2023); Ethics Committee of the Istituto Auxologico Italiano (Approval n°2022_09_27_15).

### 2.2. Measurements

The anthropometric variables considered were weight, stature, and relaxed mid-upper arm, waist and calf circumferences, measured in agreement with international criteria [26,27]. Six centers (804 individuals) followed the protocol of Lohman et al. [26], while the centers of Chieti, Florence and Padua (245 individuals) adopted the International Society for the Advancement of Kinanthropometry (ISAK) protocol [27]. As the two protocols do not differ with respect to the measurements included in this study, they were considered to be comparable. Body weight was measured to the nearest 0.01 kg using a mechanical scale (Seca scale, Seca, Hamburg, Germany; Wunder RB column scale, Wunder, Milan, Italy), and stature (distance from the vertex to the plantar surface) to the nearest 0.1 cm with a stadiometer (Seca stadiometer, Seca, Hamburg, Germany; Siber-Hegner stadiometer, GPM Anthropological Instruments, Susten, Switzerland). Circumferences were taken using an anthropometric tape (Seca medical tape, Seca, Hamburg, Germany; Lufkin tape, Apex Tool Group ATG, Sparks, MD, USA; Cescorf tape, Porto Alegre, Brazil): waist at the narrowest part of the torso; mid-upper arm with the arm relaxed, and calf at the point of maximum circumference, in a plane perpendicular to the long axis of the body segment. All measurements were performed by experienced technicians and followed the same procedures, using the same anatomical landmarks and body positioning, as indicated in both the ISAK protocol and Lohman’s reference manual [26,27]. Body mass index (BMI, kg/m^2^) was calculated as weight (kg) divided by stature squared (m^2^) [28].

The bioelectrical variables included resistance (R, Ω) and reactance (Xc, Ω), measured at 50 kHz, using single-frequency devices (BIA-101, BIA-101 Anniversary or BIA-101 BIVA^®®^ PRO) manufactured by Akern Srl (Florence, Italy). Participants were instructed to refrain from eating or drinking for at least 4 h before the measurement, to avoid strenuous physical exercise during the previous 12 h, and to abstain from alcohol consumption for at least 16 h prior to testing [29]. They were also asked to empty their bladder before the assessment and to remove any metallic objects. Before each testing session, the accuracy of the bioimpedance devices was verified using a reference circuit with known acceptance values. The bioelectrical measurements were taken using a hand-to-foot approach, with participants lying in the supine position; the legs were abducted at approximately 45° and the arms positioned about 30° away from the trunk. For the hand, a voltage-sensing electrode was placed on the dorsal surface of the wrist, between the styloid processes, while the current-injecting electrode was positioned distally, between the second and third metacarpals. For the foot, the sensing electrode was placed on the anterior surface of the ankle, and the current-injecting electrode was positioned between the second and third metatarsals [29].

### 2.3. Statistical Analyses

A priori sample size estimation was performed using the software G*Power (version 3.1.9.7). For sex- and age-based comparisons, a two-way ANOVA was considered, assuming an effect size of 0.25, a statistical power of 0.95 and a significance level of α = 0.005, resulting in a minimum required sample size of 322 participants. The final sample size substantially exceeded the calculated minimum, thus ensuring high statistical power.

The *specific* bioelectrical values, namely *specific* resistance, or resistivity (R*sp*, Ωcm) and *specific* reactance, or reactivity (Xc*sp*, Ωcm) were obtained by multiplying R and Xc by the correction factor A/L, where A is the estimated total body cross-sectional area (cm^2^) and L is the body length (cm). The total area (A, cm^2^) was estimated as 0.45×upper arm area + 0.10×waist area + 0.45×calf area. Each segment area was calculated as C^2^/4π, where C (cm) is the circumference of the relaxed mid-upper arm, waist, or calf. The body length (L, cm) was obtained as 1.1×stature [13]. The PhA was derived as the arctan of Xc/R×180/π and the *specific* impedance, or impedivity (Z*sp*, Ωcm) as (R*sp*^2^ + Xc*sp*^2^)^0.5^.

Data distribution was assessed using the Shapiro–Wilk test and visual inspection of Q–Q plots. Given the large sample size, graphical evaluation was considered in the interpretation of normality. Continuous variables were presented as mean ± standard deviation when normally distributed, and as median and interquartile range (IQR) when deviations from normality were observed.

The Pearson’s correlation coefficient (r) was calculated to assess the association between R*sp* and Xc*sp.* To investigate the main effects of sex and age and their interaction, a two-way ANOVA was performed, considering two age groups (30–49 years and 50–65 years). Effect sizes, expressed as partial eta squared (η^2^p), were calculated to quantify the magnitude of differences. According to Cohen’s guidelines [30], effect sizes were interpreted as small (η^2^p  =  0.01), medium (η^2^p  =  0.06), and large (η^2^p  =  0.14).

The new sex-specific tolerance ellipses for sub-groups of age (30–49 and 50–65 years) and combined ellipse for the 30–65 years age range were constructed. Data from the present study were then compared and pooled with previously published values for young adults [19] and older individuals [16] to create a comprehensive tolerance ellipse representing the Italian population aged 18 to 100 years. Confidence ellipses and Hotelling’s T^2^ were applied to compare groups.

To illustrate the bioelectrical peculiarities of selected conditions relative to the general sample, and to assess the applicability of the newly established reference values, a subgroup of competitive athletes engaged in strength sports, identified within the general sample, was analyzed. In addition, the distribution of individuals with pathologies characterized by important alterations of FM%, namely obesity and anorexia nervosa, was examined within the tolerance ellipses for 30–65 years. Independent samples t-tests were performed to assess differences in Z*sp* and PhA values between the groups.

Statistical analyses were performed using Jamovi (version 2.3.28) or the software *SpecificBIVA* (freely available at www.specificbiva.com). The significance threshold was set at *p* < 0.005 to reduce the risk of Type I errors, aligning the threshold to a false discovery probability of around 7%, which is close to the conventional 5%. This is in line with recommendations for increasing the reproducibility of the exposed results from substantial statistical evidence in biological sciences [31].

## 3. Results

The general population sample showed internal variability by sex and age, with two predefined age groups (30–49 and 50–65 years) characterized by median ages of 39.9 years (IQR 11.7) and 57.5 years (IQR 8.2) in women, and 37.7 years (IQR 11.5) and 58.0 years (IQR 7.8) in men, respectively (Table 1).

Effect sizes were generally small, particularly with respect to age differences and age–sex interactions.

Men had significantly greater stature, weight, BMI, and waist circumference than women. Bioelectrical values also differed significantly, with men showing higher PhA values—indicative of greater body cell mass, muscle mass and lower ECW/ICW ratio—and lower Z*sp*, corresponding to shorter vector and thus indicative of lower FM%.

**Figure 3:**
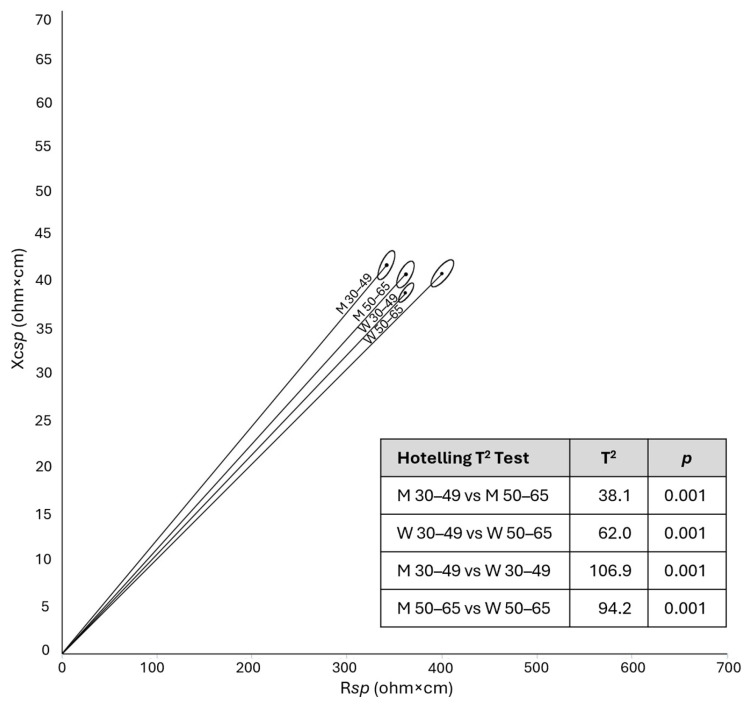
Comparison of mean bioelectrical vectors by sex and age categories, with Hotelling’s T^2^ results. Legend: M: men; W: women; R*sp*: *specific* resistance; Xc*sp*: *specific* reactance.

In both sexes, participants in the older age group showed lower stature and higher weight, BMI, waist and calf circumference than those in the younger subgroup (Table 1). They also showed a longer vector—particularly women—and a lower PhA (Table 1, Figure 3).

The differences by age were similar in the two sexes except for arm circumference and, tendentially, *specific* reactance, which were lower in older men and higher in older women compared to younger individuals. A tendency toward a significant interaction was also observed for *specific* resistance and impedance, with women showing a more pronounced age-related difference.

When the values of the two age groups were combined to represent a wider group of the Italian adult population (30–65 years), the difference between the mean bioelectrical vectors of the two sexes remained significant (T^2^: 172.2, *p* < 0.001), with men showing higher PhA and lower Z*sp* (Table 2).

**Figure 4:**
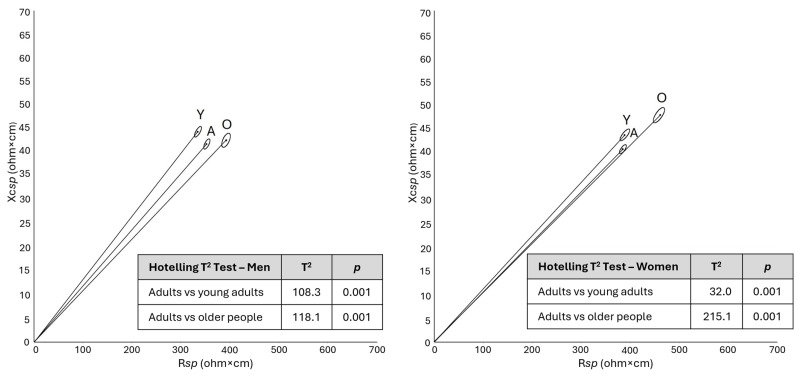
Comparison of mean bioelectrical vectors between adults (30–65 years) and other references (young and older individuals). Legend: R*sp*: *specific* resistance; Xc*sp*: *specific* reactance; A: Italian adults (current sample); O: Italian older people Y: Italian-Spanish young adults.

Consistent with internal age-related variability, the mean bioelectrical values of the whole sample differed significantly from previously published reference data for Italian-Spanish young adults [19] and Italian older adults [16] (Figure 4). Increasing age was associated with progressively lower PhA values and tendentially longer vectors. Despite these differences, Italian reference values for the population aged 18 to 100 years were defined by merging the present dataset with previously published ones on Italian individuals (Table 2).

**Figure 5:**
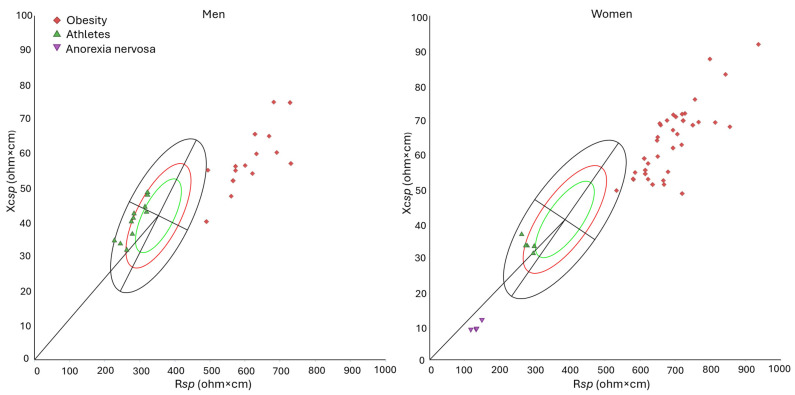
Distribution of the vectors of individuals with pathological conditions (red squares: 52 patients with obesity; purple triangles: 4 patients with anorexia nervosa) and 16 athletes (green triangles) within the tolerance ellipses for the general population from 30 to 65 years. Green, red, and black ellipses indicate the 50%, 75%, and 95% tolerance ellipses, respectively. Legend: R*sp*: *specific* resistance; Xc*sp*: *specific* reactance.

Bioelectrical vectors of competitive athletes, identified within the general sample, were predominantly positioned in the lower-left quadrant, reflecting higher phase angles (*p* < 0.001 in men, *p* = 0.045 in women) and shorter vectors (*p* < 0.001) compared with the general population (Supplementary Table S1, Figure 5).

Furthermore, the bioelectrical vectors of individuals with pathologies (obesity and anorexia nervosa; Supplementary Table S1) fell mostly outside the 95% ellipse (Figure 5). The vectors of patients with obesity, located beyond the upper pole of the ellipse, were significantly longer (*p* < 0.001) and showed lower phase angles (*p* < 0.001) than those of the general population. On the opposite side of the ellipse, women with anorexia nervosa exhibited shorter vectors (*p* < 0.001) and lower phase angles than healthy individuals (*p* < 0.001).

## 4. Discussion

The present study provided *specific* BIVA reference values derived from a healthy convenience sample of the Italian population of both sexes. Data were collected and analyzed for adults aged 30–65 years, a group not represented in the *specific* BIVA literature, and were then compared and merged with existing references to cover the full age range of 18–100 years. The sample exhibited the typical sexual dimorphism, with men showing higher phase angles and shorter vectors, and age-related differences associated with progressively lower phase angles and longer vectors. Bioelectrical vectors of individuals with obesity and anorexia nervosa fell outside the tolerance ellipses for the general population, whereas those of athletes occupied the lower left quadrant of the ellipses.

The observed patterns were coherent with theoretical expectation regarding the variability and interpretation of bioelectrical values, consistent with the established knowledge on body composition in relation to sexual dimorphism [32], ageing [33], health status [7] and physical activity [5].

### 4.1. Sexual Dimorphism

The higher PhA values of men (on average 0.7° more than in women) reflect greater muscle mass and lower ECW/ICW ratio, whereas their shorter vectors (mean difference of 32.2 Ωcm) correspond to a lower percentage of fat mass, according to *sp*BIVA interpretation. Similar sex-related bioelectrical patterns have been widely documented in the literature (e.g., [13,17,19,34,35]). In particular, the study by Mattiello et al. (2020) [17] reported clear sex differences in PhA values estimated across age groups. Except for children and adolescents (0–15 years) and people older than 80 years, where no differences were detected, men consistently showed higher values, approximately 0.8° more than women. Likewise, research on *sp*BIVA reported similarly higher phase angles, along with shorter vectors in men. Sex differences in mean vector length (Z*sp*) ranged from 55.4 Ωcm in young Italian-Spanish individuals [19] to 89.3 Ωcm in US adults [13], while were slightly lower in our sample (32.2 Ωcm), thus suggesting smaller sex-related differences.

### 4.2. Age Related Variability

Differences by age, observed both within our sample of 30–65 years and relative to previously published reference values for young adults and older people, confirmed the expected trend of increasing vector length and decreasing PhA during adulthood and ageing [19,34,35].

The longer vectors at older ages, particularly among women, indicate a trend toward a higher fat mass percentage with age. Such a finding has been consistently observed in previous research during adulthood, especially in men and during the first phases of ageing, although in very advanced ages a slowing and even a reversal of the trend has been observed in women [16,36].

Considering phase angle, participants of both sexes aged 50–65 years showed lower PhA values than those aged 30–49 years. When younger (18–30 years) and older (>65 years) groups from the literature were compared, the age-related changes in PhA showed a mean difference between the groups of 1.4° in men and 0.5° in women. These differences are similar in men and slightly lower in women to those observed by Mattiello et al. for the same age groups (1.3° in men and 1.0° in women) [17]. They reflect a gradual loss of skeletal muscle mass and quality, and of intracellular water, which are physiological changes of ageing and represent a risk for possible diseases. Indeed, PhA has been shown to be low in sarcopenic or sarcopenic obese individuals, and the prevalence of sarcopenia higher among those with low PhA [37].

### 4.3. Inter-Population Variability

To our knowledge, no *specific* BIVA reference values are available for other populations. Therefore, comparisons were limited to PhA. The newly assembled sample of 30–65 years showed mean PhA values consistent with reference data for healthy adults of similar age [34,35,38,39]. In particular, when stratified by age, our results for both sexes were highly similar compared to the percentiles provided by Campa et al. for the Italian population [38], with a maximum mean difference of 0.2°. Similar values were also reported in the global references published by Mattiello et al. [17]. Conversely, when compared with values measured in a US sample by Barbosa-Silva et al. [34], our mean phase angles were lower across sexes and age groups, with differences reaching 0.9° in young women. A similar trend toward higher PhA values (0.5°) was observed in adult Swiss women [39] and in German men [35]. These discrepancies may reflect variations in lifestyle factors (dietary patterns and physical activity levels) and genetic background among the populations studied, together with methodological differences related to the use of different devices across the studies mentioned above.

### 4.4. Illustrative Applications

The new reference values offer potential applications in the fields of clinical practice, nutritional studies and sports science. Indeed, the illustrative examples of athletic and pathological cases showed peculiar differences from the general population: the bioelectrical vectors of athletes occupied the left-lower quadrant of the ellipse while those of individuals with obesity and anorexia nervosa fell outside the 95% variability.

The results are consistent with the theoretical expectations of *sp*BIVA: the bioelectrical vectors of competitive athletes, engaged in bodybuilding, streetlifting and tennis, were located on the left-lower side of the ellipse, consistent with the expected low ECW/ICW ratio and high body cell and muscle mass [40], although remaining within the range of the physiological variability of the general population. Their elevated PhA values, on average 8.1° in males and 6.8° in females, align with previous observations in bodybuilders: Piccoli et al. [41] reported mean PhA of 8.6° in men, while Giakoni-Ramírez et al. [42] found mean PhA values of 8.2° in men and 7.0° in women. However, our values were lower than those reported by Nunes et al. [43] (9.3°) in men. Overall, in sports science, the availability of the new reference ellipses allows athletes to be compared to the variability of Italian adults as well as across disciplines within the same reference framework.

Patients with obesity clustered in a well-defined region of the graph, substantially exceeding the 95% tolerance ellipse at its upper right pole. These individuals were characterized by long vectors, indicative of their high amounts of fat mass [13], and by low PhA, likely reflecting the downward trend of the association between PhA and body fat, observed in people with obesity [44]. Conversely, individuals with anorexia nervosa, characterized by extremely low body weights, were positioned in the lower pole of the ellipse, far outside the 95% tolerance region. Notably, in these cases, regression equations for estimating fat mass in patients with anorexia nervosa [45] yielded negative absolute values [24]. Interestingly, the mean vectors of pathological groups fell outside the 95% tolerance ellipse, even when compared with the overall tolerance ellipse for Italian individuals aged 18 to 100 years, indicating that these extreme conditions lie beyond the physiological variability of the general population, regardless of age-related differences. This result has both theoretical and methodological implications, as it highlights the overwhelming effect of pathology on body composition variability and allows the use of a broad age-range reference as a practical benchmark for health-related applications.

In summary, this study has the strength to address a gap in the literature by providing reference values for adults aged 30–65 years and enabling the generation of comprehensive values from 18 to 100 years, applicable for body composition evaluation, comparative purposes and for clinical applications. The use of standardized procedures, the same anthropometric landmarks, and the same device type and manufacturer further strengthen the study by ensuring the reliability and comparability of the data. However, some limitations should be acknowledged. The relatively small size of the athlete subgroups and of the independent sample of patients with anorexia nervosa included for comparative purposes represents a limitation of the present study. Furthermore, the references have been derived in a convenient sample of persons from the general population included without using a specific sampling procedure that would have made it fully representative of the Italian population. Indeed, the sample was primarily drawn from central and northern Italy, underscoring the need for further research to achieve more balanced coverage of the national population. Future work may also focus on investigating longitudinal changes. Indeed, from a clinical perspective, tracking shifts in *specific* vectors through serial measurements can be used for monitoring the efficacy of interventions. The role of sociodemographic characteristics within the study population, as well as the variability across other populations, represent further targets for research.

## 5. Conclusions

The new *specific* tolerance ellipses for the Italian adult population aged 30–65 years show the expected pattern of sex- and age-related variation. Bioelectrical vectors of individuals with pathological body composition abnormalities (obesity and anorexia nervosa) were outside the 95% range of variability. Athletes’ vectors also show a peculiar position, thus highlighting the possibility to analyze body composition features with respect to the general population and across different sports.

These reference values fill a gap in the existing literature, providing an updated framework for interpreting body composition and for conducting comparative analyses in adults, thereby supporting clinical, public health, and sports science research and applications.

Stratification by age group enables focused comparisons, whereas the full 18–100 age range allows direct comparisons of individuals or groups across different ages and facilitates the monitoring of pathological conditions regardless of age.

## Figures and Tables

**Table 1:** Descriptive and comparative statistics of the sample by sex and age group.

	Men				Women				Two-Way ANOVA, Effect of:					
	30–49 Years (*n* = 210)		50–65 Years (*n* = 231)		30–49 Years (*n* = 248)		50–65 Years (*n* = 360)		Sex		Age		Interaction	
	Mean	SD	Mean	SD	Mean	SD	Mean	SD	*p*	η^2^p	*p*	η^2^p	*p*	η^2^p
Stature (cm)	174.0	7.6	173.0	7.7	162.0	6.7	160.0	7.2	<0.001	0.418	<0.001	0.016	0.929	0.000
Weight (kg)	75.7	11.8	78.0	12.2	60.3	10.0	63.7	11.8	<0.001	0.286	<0.001	0.015	0.466	0.001
BMI (kg/m^2^)	24.8	3.2	26.2	3.6	23.0	3.7	24.9	4.3	<0.001	0.039	<0.001	0.041	0.274	0.001
Waist crf (cm)	84.6	9.5	91.5	10.5	72.8	9.0	80.1	10.6	<0.001	0.244	<0.001	0.108	0.837	0.000
Arm crf (cm)	31.0	3.3	30.5	3.2	27.2	3.5	28.6	3.5	0.027	0.145	<0.001	0.005	<0.001	0.017
Calf crf (cm)	36.8	2.6	37.1	3.3	35.2	3.6	35.6	3.6	0.124	0.050	<0.001	0.002	0.633	0.000
R*sp* (Ωcm)	342.0	53.1	361.0	56.3	362.0	60.2	400.0	75.3	<0.001	0.049	<0.001	0.045	0.017	0.005
Xc*sp* (Ωcm)	42.3	9.3	41.3	8.9	39.3	8.1	41.4	9.4	0.010	0.006	0.316	0.001	0.008	0.007
Z*sp* (Ωcm)	345.0	53.5	364.0	56.6	364.0	60.6	402.0	75.6	<0.001	0.047	<0.001	0.044	0.016	0.006
PhA (°)	7.0	1.0	6.5	1.0	6.2	0.8	5.9	0.9	<0.001	0.132	<0.001	0.042	0.052	0.004

BMI: body mass index; crf: circumference; R*sp*: *specific* resistance; Xc*sp*: *specific* reactance; Z*sp*: *specific* impedance; PhA: phase angle; n: sample size; SD: standard deviation; η^2^p: partial eta squared.

**Table 2:** Overview of the *specific* bioelectrical reference values for the Italian population across sexes and age groups, based on data from the literature and the present study.

Age Group	Sex	Sample Size	R*sp* (Ωcm)		Xc*sp* (Ωcm)		PhA (°)		r R*sp*, Xc*sp*	Reference
			Mean	SD	Mean	SD	Mean	SD		
18–30 years	M	103	334.3	36.7	43.6	6.6	7.4	0.9	0.75	Ibáñez et al., 2015 [19] *
	W	117	394.6	65.6	42.8	8.2	6.2	0.7	0.83	
30–49 years	M	210	342.0	53.1	42.3	9.3	7.0	1.0	0.70	Current study
	W	248	362.0	60.2	39.3	8.1	6.2	0.8	0.79	
50–65 years	M	231	361.0	56.3	41.3	8.9	6.5	1.0	0.67	Current study
	W	360	400.0	75.3	41.4	9.4	5.9	0.9	0.76	
30–65 years	M	441	352.3	55.5	41.8	9.1	6.8	1.0	0.67	Current study
	W	608	384.9	71.2	40.7	9.4	6.1	1.0	0.72	
65–100 years	M	265	391.8	57.9	42.6	9.9	6.2	1.2	0.59	Saragat et al., 2014 [16]
	W	295	462.0	80.1	47.9	11.2	5.9	1.0	0.75	
18–100 years	M	809	363.0	58.1	42.2	8.9	6.7	1.1	0.62	Pooled data
	W	1020	408.4	81.1	42.9	9.9	6.0	0.8	0.80	

Legend: M: men; W: women, R*sp*: *specific* resistance; Xc*sp*: *specific* reactance; SD: standard deviation; r: Pearson’s correlation coefficient. * Only Italian participants were considered.

## Data Availability

Dataset available on request from the corresponding authors due to restrictions related to the inclusion of human participants. Access is limited to research purposes consistent with ethical standards.

## Supplementary Materials

The following supporting information can be downloaded at: https://www.mdpi.com/article/10.3390/jfmk11010081/s1, Table S1: Anthropometric and bioelectrical characteristics of pathological samples and athletes.
