# Supplementary material for: Specific Bioelectrical Vector Reference Values for Italian Adults: A Multicentre Study
Source: J Funct Morphol Kinesiol. 2026 Feb 17;11(1):81. doi: 10.3390/jfmk11010081 (PMC12921988; doi:10.3390/jfmk11010081)
Supplement: Supplementary file 1 [file jfmk-11-00081-s001.zip › jfmk-4114627-supplementary.pdf]

Supplementary Table S1. Anthropometric and bioelectrical characteristics of pathological samples and athletes

Group	Men				Women					
	Obesity (n=15)		Athletes* (n=11)		Obesity (n=37)		Anorexia (n=4)		Athletes* (n=5)	
	Mean	SD	Mean	SD	Mean	SD	Mean	SD	Mean	SD
Age (years)	53.5	10.4	34.7	4.9	51.8	11.5	32.5	12.7	37.3	5.4
Height (cm)	176.0	4.8	164.9	12.2	162.6	5.5	166.3	9.0	163.6	3.4
Weight (kg)	118.7	10.9	70.0	9.0	95.9	14.9	31.7	2.8	58.2	1.9
BMI (kg/m^2)	38.4	4.2	24.2	1.9	36.3	5.7	11.5	0.9	21.8	0.7
Waist crf (cm)	127.6	11.1	76.0	4.1	111.8	12.2	51.4	0.9	67.9	3.7
Arm crf (cm)	39.9	3.5	32.3	3.0	39.3	3.6	14.7	0.9	26.6	1.3
Calf crf (cm)	45.5	3.5	34.7	3.5	45.0	4.2	23.9	1.1	35.6	1.0
R*sp* (Ωcm)	615.7	75.2	284.7	32.2	691.0	84.3	195.3	21.7	281.5	15.3
Xc*sp* (Ωcm)	58.3	9.2	40.5	5.6	63.7	10.6	13.8	2.2	33.4	2.0
Z*sp* (Ωcm)	618.4	75.5	287.6	32.6	694.0	84.7	196.1	21.8	283.5	15.0
PhA (°)	5.4	0.6	8.1	0.5	5.3	0.5	4.0	0.3	6.8	0.7

Legend: BMI: body mass index; crf: circumference; R*sp*: *specific* resistance; Xc*sp*: *specific* reactance; Z*sp*: *specific* impedance; PhA: phase angle; SD: standard deviation

*Data refer to elite strength athletes, identified within the general sample, engaged in bodybuilding, streetlifting, and tennis
